# Supplementary material for: Dye-cycling DNA origami rotors for long-term tracking of transcription at base-pair resolution
Source: Cell Rep Methods. 2026 Aug 13;6(10):101550. doi: 10.1016/j.crmeth.2026.101550 (PMC13721745; doi:10.1016/j.crmeth.2026.101550)
Supplement: Supplementary file 1 [file mmc1.pdf]

Cell Reports Methods, Volume *6*

Supplemental information

Dye-cycling DNA origami rotors for long-term tracking of transcription at base-pair resolution

Amanda L. Wacker, Ryan J. Fantasia, Brian Tenner, Boyu Liu, Jerry D. Wu, Nicholas Monell, and Pallav Kosuri

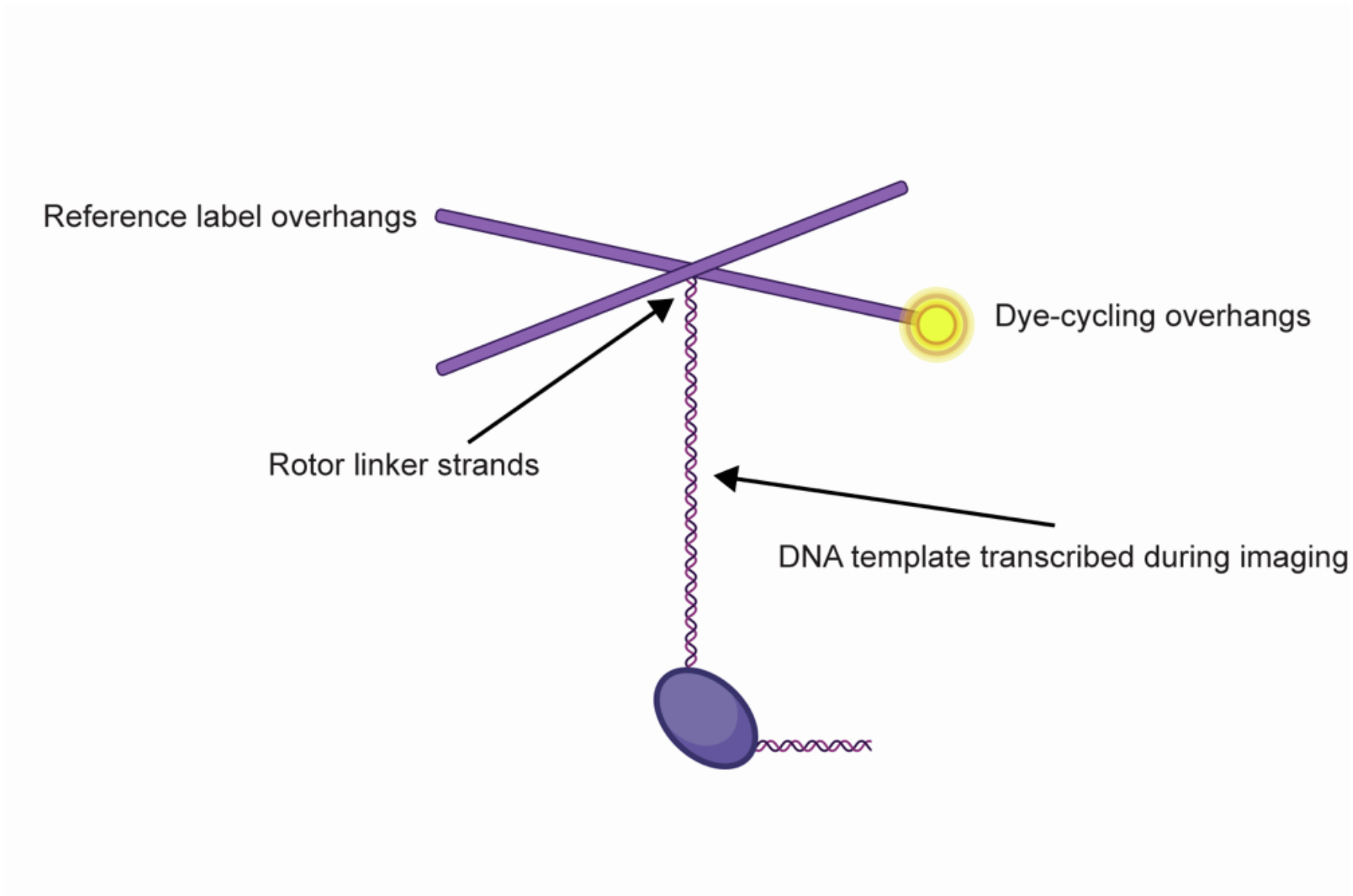

Figure S1. dcORBIT construction, related to STAR methods | Schematic of dcORBIT indicating locations of relevant DNA sequences. Not to scale.

$$OP^* = \frac{1}{1 + \frac{k_{bleach} + k_{off}}{k_{on}} + \frac{k_{bleach}}{k_{off}}}$$

Model

Description	Variable	Equation
Overhang	$O(t)$	$\frac{dO}{dt} = k_{off}OP^* + k_{off}OP - k_{on}O$
Overhang w/ Probe	$OP^*(t)$	$\frac{dOP^*}{dt} = -k_{off}OP^* - k_{bleach}OP^* + k_{on}O$
Overhang w/ Bleached Probe	$OP(t)$	$\frac{dOP}{dt} = -k_{off}OP + k_{bleach}OP^*$

Parameters

Description	Parameter
On rate (1 nM *P**)	k_{on}
Off rate	k_{off}
Bleach rate	k_{bleach}

Assumptions

Description	Assumption
Concentration of probes is large	$\frac{dP^*}{dt} = 0, k_{on} = kP^*$
Total binding sites are conserved	$O_{total} = O + OP^* + OP$

Figure S2. Dye-cycling kinetic model, related to Figure 1 | a Dye-cycling model equation used to optimize probability of an overhang being bound by a probe that is able to emit fluorescence.

Probe Name	Probe Sequence	k_{on} [1/s • nM]	k_{off} [1/s]
7A	5cy5/AGGAGGA	0.17	0.40
7G	5cy5/GAGGAGG	0.27	0.55
8	5cy5/GAGGAGGA	0.07	0.08
9	5cy5/GAGGAGGAG	0.07	0.05

Supplemental Table 2. Dye-cycling probe parameters, related to STAR methods | Dye-cycling probe sequences and experimentally measured parameters. 7A k_{on}: n = 75; 7A k_{off}: n = 79; 7G k_{on}: n = 62; 7G k_{off}: n = 75; 8 k_{on}: n = 72; 8 k_{off}: n = 75; 9 k_{on}: n = 74; 9 k_{off}: n = 101.

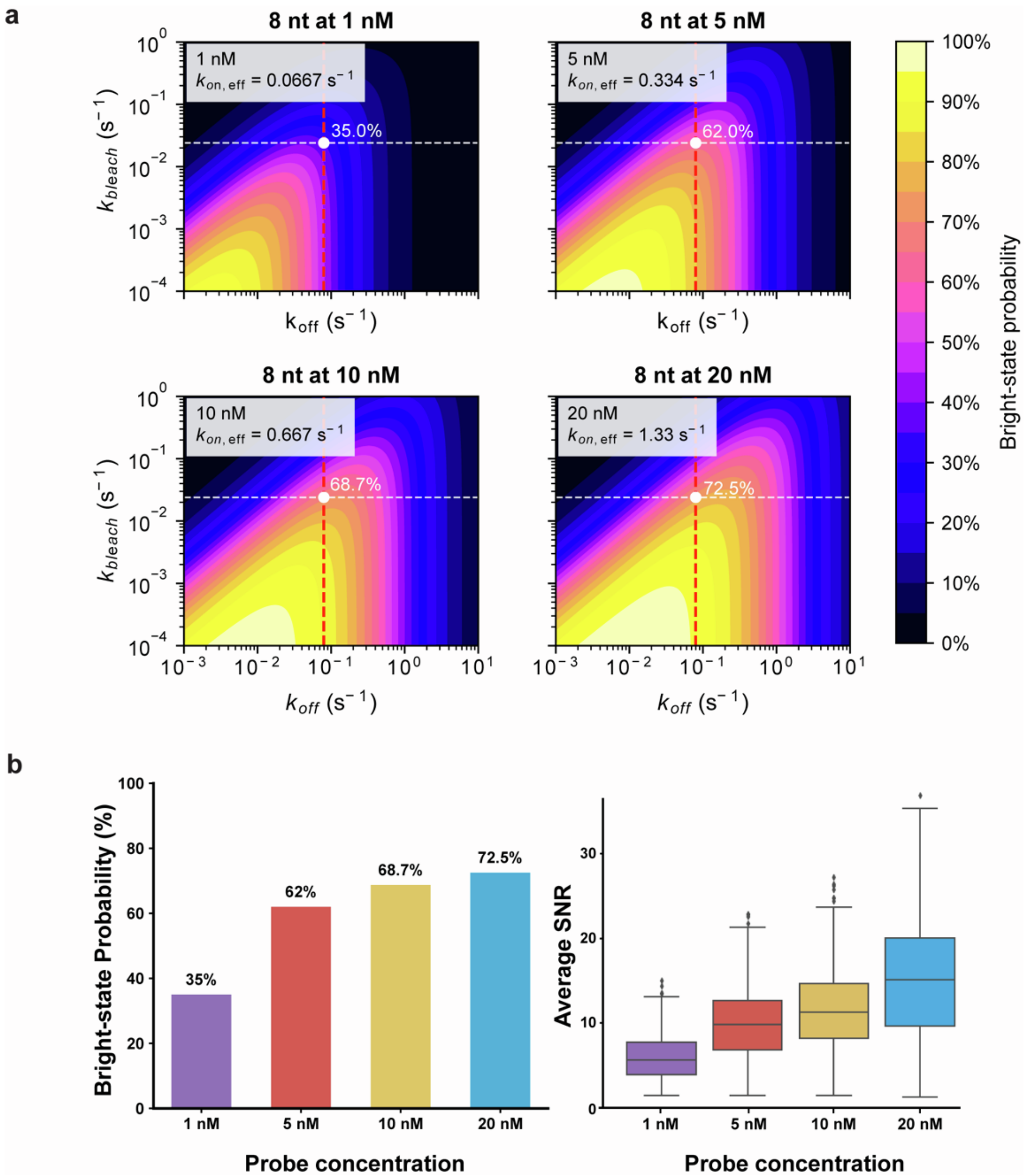

Figure S3. Application of dye-cycling kinetic model, related to STAR methods and Table S2 | a Bright-state (*OP**) probability landscapes using rates using probe "8" (8 nt) from Supplemental Table 2 and dye cycling kinetic model described in Figure S2. **b** *Left*: Bright-state probabilities calculated for the probe concentrations as in (a). *Right*: Experimental average SNR for dye-cycling rotors using 8 nt probe at different concentrations measured in a ten-minute recording.

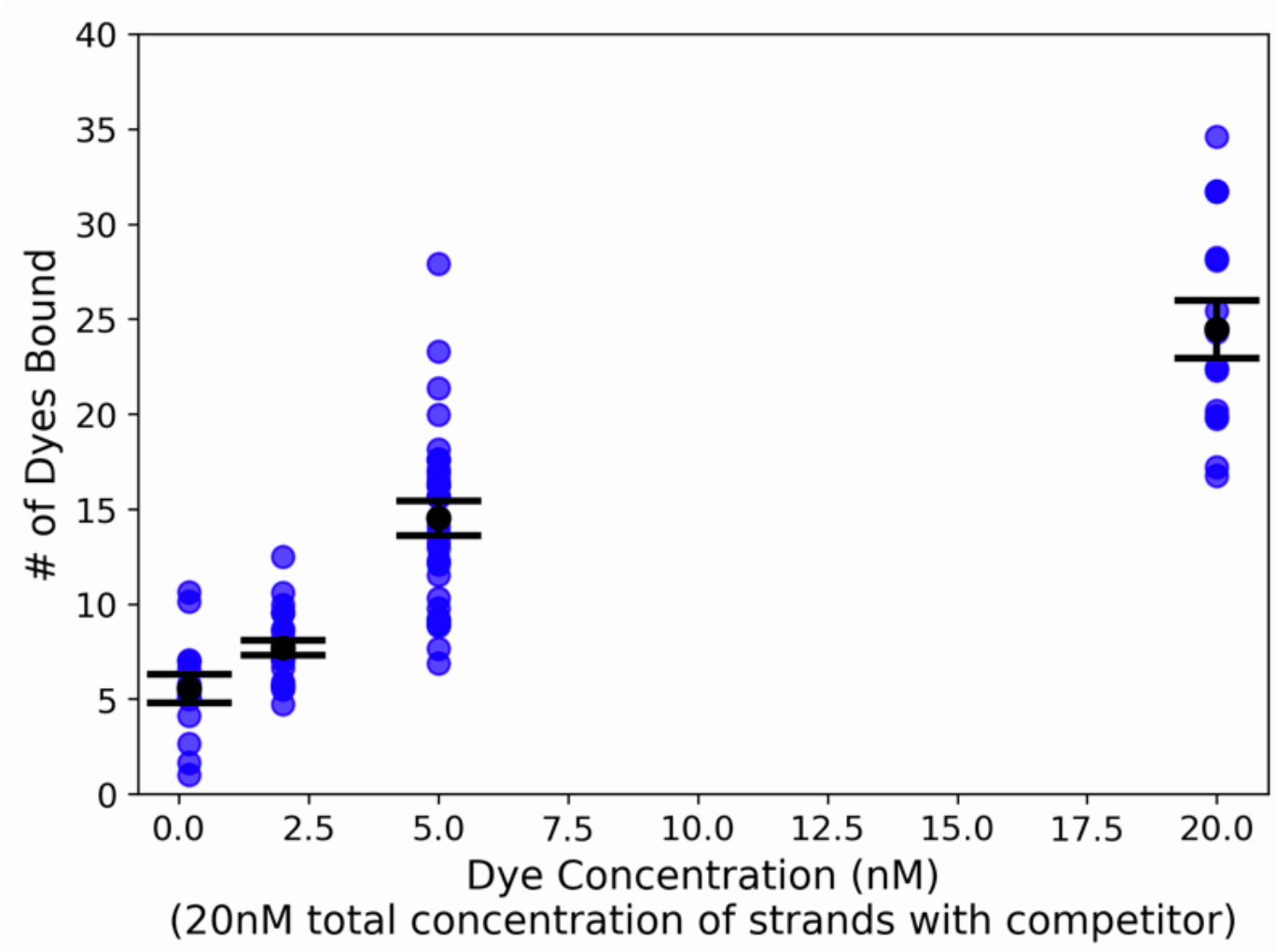

Figure S4. Estimation of number of dyes bound during dye-cycling, related to STAR methods| Measurement of number of bound dyes at different concentrations of probes in solution. To accurately estimate the number of dyes bound per rotor in our experiments, we performed a series of experiments with different concentrations of probes in solution. We also measured the average intensity of a single dye as a reference. To determine the number of dyes bound, we measured the fluorescent intensity of individual rotors on the first frame of each recording, subtracted the local background signal, and divided the resulting intensity by the average fluorescent intensity of a single dye. Blue dots represent calculations for a single rotor at that condition. 200 pM fluorescent probe: *n* = 13; 2 nM fluorescent probe: *n* = 22; 5 nM fluorescent probe: *n* = 29; 20 nM fluorescent probe: *n* = 14.

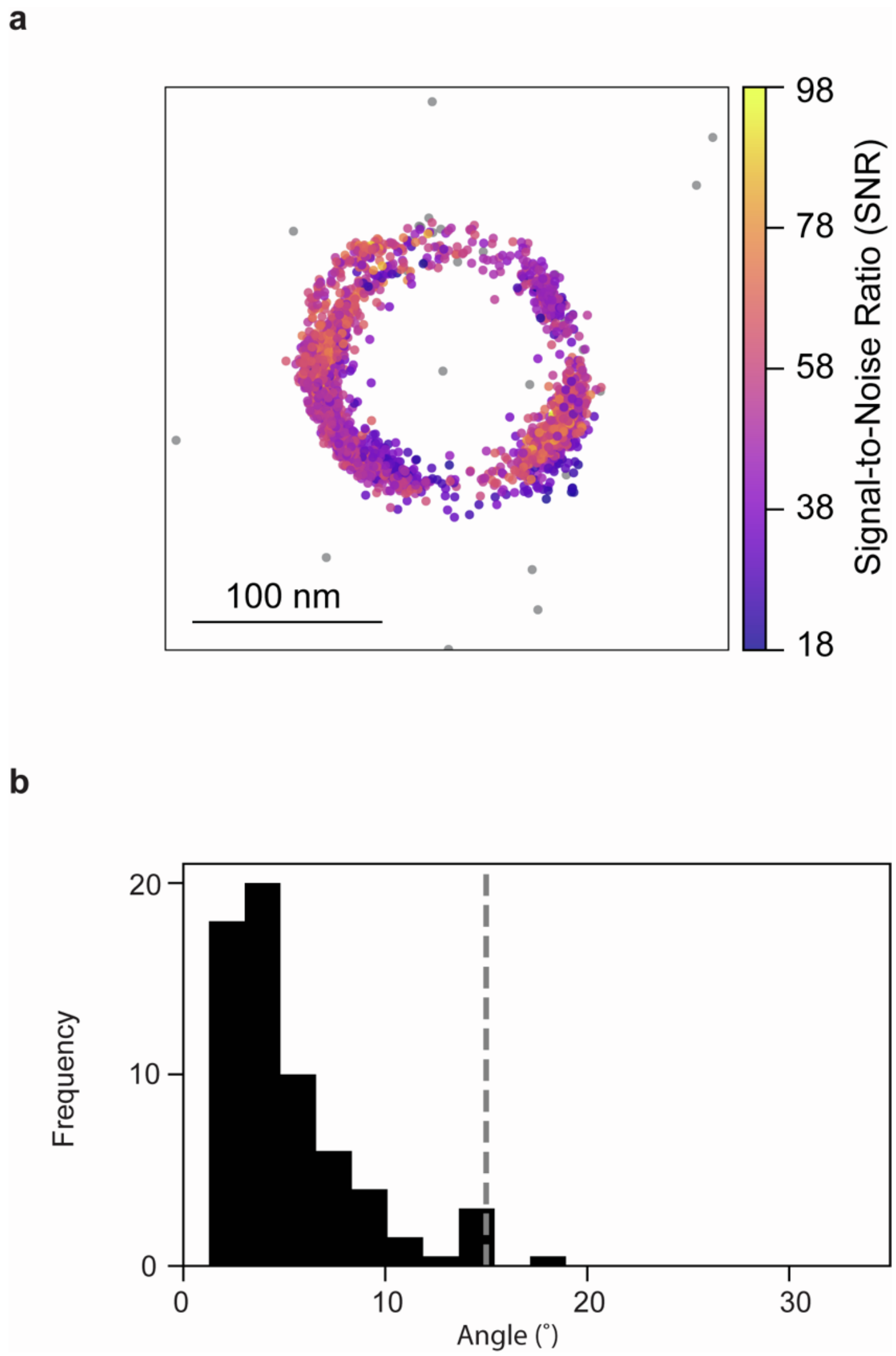

Figure S5. Quality control of localizations and step measurements, related to STAR methods | a Example rotor localization during dye-cycling recording colored based on Signal to Noise Ratio (SNR) threshold. Observations with SNR less than 18 colored in grey. Scale bar: 100 nm. **b** Histogram standard error of measurement of unfiltered forward step size measurements. Gray vertical line indicates 15°; the standard deviation of *n* = 127 unfiltered step measurements.
